# Factors Influencing the Incidence of Obesity in Australia: A Generalized Ordered Probit Model

**DOI:** 10.3390/ijerph14020177

**Published:** 2017-02-11

**Authors:** Gulay Avsar, Roger Ham, W. Kathy Tannous

**Affiliations:** School of Business, Western Sydney University, Parramatta, NSW 2150, Australia; g.avsar@westernsydney.edu.au (G.A.); rogham@gmail.com (R.H.)

**Keywords:** obesity, panel model, generalized ordered probit

## Abstract

The increasing health costs of and the risks factors associated with obesity are well documented. From this perspective, it is important that the propensity of individuals towards obesity is analyzed. This paper uses longitudinal data from the Household Income and Labour Dynamics in Australia (HILDA) Survey for 2005 to 2010 to model those variables which condition the probability of being obese. The model estimated is a random effects generalized ordered probit, which exploits two sources of heterogeneity; the individual heterogeneity of panel data models and heterogeneity across body mass index (BMI) categories. The latter is associated with non-parallel thresholds in the generalized ordered model, where the thresholds are functions of the conditioning variables, which comprise economic, social, and demographic and lifestyle variables. To control for potential predisposition to obesity, personality traits augment the empirical model. The results support the view that the probability of obesity is significantly determined by the conditioning variables. Particularly, personality is found to be important and these outcomes reinforce other work examining personality and obesity.

## 1. Introduction

One in four Australian adults was obese in 2009 with another one-third being overweight [1]. Over the last two decades, there has been a steady shift in the Australian population towards the higher end of the body mass index (BMI), driven mainly by weight gain rather than by changes in height. The BMI, a simple index of weight for height, is commonly used to classify people as overweight and obese. It is defined as the weight in kilograms divided by the square of the height in metres (kg/m^2^) [2]. An Australian study suggests that excessive body weight is likely to be costly, with an estimated economic cost including direct health costs, productivity losses, and carer costs of Australian $60 billion dollars per year [3]. The increasing prevalence of obesity is linked to the onset of chronic diseases including type 2 diabetes, hypertension, coronary heart disease, elevated cholesterol levels, depression, and musculoskeletal disorders [4,5,6,7]. Other studies have demonstrated that obesity is associated strongly with a deterioration in health-related quality of life, including both the physical and mental health domains [8]. It has also been demonstrated that obesity negatively affects workforce participation and gives an increased risk of occupational injury [9,10,11]. This has resulted in a growing demand for research to better understand the factors that determine obesity [12] and the socio-economic impact of being overweight. 

This paper explores those factors that influence the incidence of obesity among Australians by way of a random effects generalized ordered probit model. The paper utilizes data from the Household Income and Labour Dynamics in Australia (HILDA) Survey, a household-based annual panel survey. The HILDA is a survey of Australian representative households with an aim to provide longitudinal data on households and their members. The same households and their members are interviewed every year. It began in 2001 with a national probability sample of 7682 households, comprising 13,969 persons interviewed (aged 15 and over) and 4784 children under age 15. It has sample retention of approximately 95% from year to year. It also has new households formed from household members that split-off, such as children leaving home or couples separating [13]. 

A component of the survey is a self-completion questionnaire (SCQ) that is provided to all individuals in the households aged 15 and over. Since 2006, the SCQ included additional questions on the height and weight of the individuals, with the individuals self-reporting the information [14]. This enables the calculation of a BMI score for each person, from which can be derived a categorical variable based on World Health Organization (WHO) guidelines. It is recognized that BMI is an imperfect measure of obesity and does not take into account sex, age, fat distribution, or muscle mass [15]. However, for HILDA respondents, it is the available variable, and, while it is an indirect measure of weight, it has been determined to correlate well with direct measures such as dual-energy X-ray absorption [15]. 

This paper comprises an examination of the influence of economic and social factors on the probability of an individual being in the WHO *overweight* and *obese* categories. The conditioning variables are demographic, economic, social, and lifestyle related, and many have been included in studies elsewhere [16,17,18]. A potential issue is that such studies may ignore a predisposition towards overweightness/obesity, which might confound the potential relationships with the candidate covariates. One way to capture this predisposition is through the use of a latent class model [16,19]. Alternatively, it could be argued that any predisposition to obesity may be captured by variables identifying personality traits. Sullivan et al. argue that personality traits can influence diet and therefore may be important in determining the propensity to obesity [20]. This study examines the influence of personality traits on the incidence of obesity. The HILDA collected data in waves five (2005) and nine (2009), from which factor scores for the five factor model (FFM) personality traits were computed. Personality traits have been identified to have a multifaceted impact on body weight [21]. The five traits are emotional stability (known for its antithesis, neuroticism), extroversion, openness to experience, agreeableness, and conscientiousness [22]. Low scores on emotional stability, thereby high scores for neuroticism, by individuals mean that they often experience feelings of anxiety, hostility, worry, and depression [21,23,24]. These individuals have then been found to weigh more and are at greater risk of obesity [24,25], with an odds ratio of 1.02. Openness is intellectual curiosity, the need for variety, and willingness to explore new things [21,26]. In Jokela et al., a meta-analysis determined that higher openness to experience was associated with slightly lower odds of obesity but this disappeared when adjusted for education (odds ratio 0.95) [27]. Extraversion is the sensitivity to positive emotions and social assertiveness [28]. Higher extraversion was not identified with obesity in women but was in men (odds ratio 1.09) in European samples, although not in America [27]. The agreeableness trait describes individuals who demonstrate trust, altruism, and generosity [28]. In Jokela et al., agreeableness was not identified with obesity (odds ratio 1.02) [27]. Individuals with conscientiousness prefer planned rather than spontaneous behaviour [28]. They were determined to maintain healthy weight by seeking healthy eating habits [18,21,27]. Jokela et al. determined high conscientiousness was associated with lower obesity risk, with an odds ratio of 0.84. In addition, Jokela et al. demonstrated a likelihood of reversion to being non-obese among initially obese individuals after 5.4 years (odds ratio 1.09) [27]. These five personality scores are included in this paper as conditioning variables in the probability model.

The next section deals with the empirical model and how it is used to test for those factors that influence obesity. A third section gives a description of the data used in the paper, along with summary statistics. This is followed by an analysis of the estimates and tests, and the paper ends with some concluding remarks. 

## 2. The Econometric Model

Obesity is usually described in terms of an ordered response model, in which the underlying latent variable is the BMI score [16,19,29]. 

For the ordered responses, the outcome for a categorical response variable is defined as:

(1) $$y_{i}=\;0,\;1,\;\ldots ,J\left(J>2\right)$$

where the *J* outcomes have a natural integer ordering. Further, a latent variable (in this case BMI score), which underlies the response variable, is defined as [30] (p. 655):

(2) $$y_{i}^{*}=x_{i}^{\prime }\beta +\varepsilon_{i}$$

where the variables in vector **x** are seen to govern the ordered responses of individuals and, for identification, do not contain a constant. The observed responses can be associated with the underlying latent variable (in this case BMI):

(3) $$y_{i}=j\Leftrightarrow \gamma_{j-1}<y_{i}^{*}\leq \gamma_{j}y_{i}\;(j=1,2,\;...,J)$$

where the *γ_j_* are thresholds or cut points to be estimated.

The response probability is given by [31] (p. 520):

(4) $$p\{y_{i}=j|x_{i}\}=p\{\gamma_{j-1}<y_{i}^{*}\leq \gamma_{j}|x_{i}\}=F(\gamma_{j}-x_{i}^{\prime }\beta )-F(\gamma_{j-1}-x_{i}^{\prime }\beta )\;(j=1,2,\;...,J)$$

with the restriction that $\gamma_{0}=-\infty$ and $\gamma_{j}=\infty$. The function *F* is an appropriate cumulative distribution function for $\varepsilon_{i}$.

Greene et al. cogently argue that, when modelling BMI category outcomes where those categories are rigidly bounded by WHO guidelines, it might be more appropriate to model ordered responses with flexible boundaries, allowing for sources of individual heterogeneity in terms of the relationship between well-being and BMI category [16,19]. 

In the generalized ordered response model, the thresholds are not fixed (parallel), but are allowed to vary across individuals. Individual heterogeneity is captured by allowing thresholds to vary with those variables that condition category probability [32]. That is:

(5) $$\gamma_{ij}={\tilde{\gamma }}_{j}+x_{i}^{\prime }\delta_{j}$$

Substitution of Equation (5) into the cumulative distribution of Equation (4) gives [33]:

(6) $$p\{y_{i}\leq j|x_{i}\}=F({\tilde{\gamma }}_{j}+x_{i}^{\prime }\delta_{j}-x_{i}^{\prime }\beta )=F({\tilde{\gamma }}_{j}-x_{i}^{\prime }\beta_{j})$$

where **β***_j_* = **β** − **δ***_j_*, leading to a separate set of coefficients for each category. The generalized model of Equation (6) is estimated as a series of *J* − 1 binary response models [34], proceeding sequentially on the series from the first model, which analyses category 1 versus 2, ..., *J*, to the last model, which analyses category 1, ..., *J* − 1 to *J*. 

In panel random effects, individual heterogeneity is also introduced by augmenting Equation (6) with the mean zero and constant variance *σ*^2^_α_ variable *α_i_*. That is, the latent variable is specified as [30] (p. 662):

(7) $$y_{it}^{*}=x_{it}^{\prime }\beta +\alpha_{i}+\varepsilon_{it}$$

leading to the cumulative distribution function [35]:

(8) $$p\{y_{i}\leq j|x_{i}\}=F({\tilde{\gamma }}_{j}-x_{i}^{\prime }\beta_{j}-\alpha_{i})$$

where individual heterogeneity is captured by the non-parallel cut offs and the panel random effects component.

Conditional on $\varepsilon_{it}|x_{i},\alpha_{i}\sim N(0,1)$, in Equation (7), we estimate a random effects generalized ordered probit for a three category variables based on individual BMI scores for the last five years of the HILDA survey. The three ordered categories, based on the WHO guidelines, are *normal*, *overweight* and *obese*. The generalized model nests alternative models based on restricting the parameters to be identical between categories. Clearly the most specific model is the standard ordered probit, in which all parameters are identical between categories. We adopt a sequential procedure advocated by Pfarr et al., following Williams, in testing down from the generalized model [34,35]. In the first round, a Wald test is performed on the restriction that all parameters are the same across categories. The model is then re-estimated with the restriction that the least significant parameter in the first round is identical across all categories. The Wald test is then applied again. This process of estimation, testing restrictions, and then applying a restriction to a new estimate proceeds until only parameters that are significantly different over categories remain. The model was estimated using REGOPROBIT2 (Statistical Software Components, Chestnut Hill, MA USA) [33].

## 3. Data and Descriptive Statistics

All data come from the HILDA panel. It needs to be noted that some of the data were imputed following non-response by a panel member or the failure of a household to provide some information. Imputation for different data items, such as income, is undertaken by making use of responses from similar individuals or households [13]. For this paper, the data were further reduced by the researchers, with missing responses for key variables being dropped to ensure balance.

Table 1 presents the list of variables included in the model. According to WHO international classifications, the BMI cut-off points for adults are less than 18.5 for *underweight*, range between 18.5 to 25.0 for *normal* weights, between 25 to 30 for *overweight* and more than 30 for *obese*. The category *underweight* is not considered in the dependent variable *ordobese*, since our analysis focuses on the *overweight* and *obese* categories relative to the normal category of BMI. This paper also uses BMI categories and not the BMI numerical values. The use of BMI categories has been criticized due to a reliance, when calculating BMI, on self-reported height and weight and the possibility of misstatement [17,18,36]. In addition, the use of categories of BMI and not BMI numerical values results in a loss of information. These criticisms are duly noted with recognition, as per Greene et al., that the BMI category is likely to be correct [16,18]. The correlation coefficient was determined to be very high for self-reported weight and height and measured weight and height (greater than 0.9) [37]. In addition, policy-makers are interested in categories and individual movements in the categories rather than the marginal changes in them [16]. 

*Age* is expected to have a quadratic association with BMI [38]. The general increasing trend of BMI with age may be attributed to age-related losses in lean body mass, resulting in lowered energy expenditure. However, later in life, BMI is expected to decrease with age due to biological mechanisms. To account for this pattern in the relationship between age and BMI, age and age squared terms are included in the model presented in this study.

A significant relationship between education and obesity has been shown in many studies [39,40]; those with higher levels of education have a significantly lower risk of obesity. The variable *educ*, the self-reported highest level of education attained by participants, was included in this model to capture this relationship and was collapsed into four categories, as outlined in Table 1.

The respondent’s employment status, *empstatus*, was re-coded as a binary variable, scoring 1 for employed and 0 for unemployed or not being in the work force. Income is captured by the variable *lndinc/p*, which is the logarithm of the ratio of household annual disposable income to the number of persons in the household who were included in the survey at the time the data was collected. The covariate *losat*, satisfaction with life, was collapsed from ten to three categories, especially to re-categorize those who rated themselves as dissatisfied with their life.

The covariates *area,* remoteness area, and *advantage*, the SEIFA 2001 decile of index of relative socio-economic advantage/disadvantage, are included in this model to capture a likely association between living in remote areas and being in relatively low socio-economic status with a higher overweight risk. Consumption spending on alcohol (*alcohol*) and on foods prepared outside the home (*meals*) is generally associated with increased obesity. Potential differences in terms of varying household types are captured by the inclusion of the variables *marstatus* and *hhtype*, where the latter is designed to control for single parents.

The five personality traits associated with the FFM are included as measures of the health status and personalities of respondents. The panel on obesity runs from 2006 to 2010 inclusive. Personality data was collected for the years 2005 and 2009. Personality scores are relatively stable [41], and scores for the year 2005 were applied to the years 2006 and 2007, while scores for 2009 were applied to the years 2008 and 2010 to complete the panel. This technique is in keeping with Cobb-Clark and Schurer in their study of the FFM from HILDA and their demonstration that personality traits are stable for working-age adults [41]. The FFM is well established in psychology literature [42] but is used less frequently in econometric work [43]. HILDA respondents are administered a version of the Big Five personality inventory, based on Saucier (1994), using the trait descriptive approach [44]. Respondents were asked how well 36 different adjectives describe them, with 28 used to derive scales of five specific personality traits. Scores for each of the traits are constructed by assigning a value from 1 to 7 to each item, with the higher score indicating that the trait describes the individual better, summing them, and obtaining an average [22,41,42]. The five personality traits are *opene*, openness to experience; *consc*, conscientiousness; *extrv*, extraversion; *agree*, agreeableness; and *emote*, emotional stability. The internal reliability coefficients (Cronbach alpha) for these traits were shown by Wooden to be satisfactorily high in HILDA (greater than 0.7) and identical between wave five and wave nine of the survey [22]. Testing was conducted by Wooden on the extent to which these personality traits changed by age between the two survey years (2005 and 2009) to conclude that for those aged 25 to 64, the personality scores for most individuals do not change much over time [22]. Some work has been done linking personality traits and obesity [20,45,46]. These studies use different personality variables to the FFM; the former uses the Karolinska Scales of Personality (KSP), and Sullivan et al. use the Temperament and Character Inventory (TCI). Fortunately, the TCI can be linked to the FFM [47].

The mean and standard deviation scores for the continuous variables in Table 1, *age*, *lndinc/p*, *alcohol*, and *meals*, have their usual meaning. The personality scores are ordinal but take on 36 different ranks between the values 1 and 7 inclusive; as such, the reported means and standard deviations have the usual interpretation. The mean for the binary variables, *gender*, *empstatus*, *hhtype*, and *marstatus*, give the proportion of the estimation sample scoring 1. The means and standard deviations for the remaining variables, which are all ordered categorically, should be interpreted with caution. The relative frequency distributions for these categorical variables are given in Table 2.

In Table 2, *advantage* is the SEIFA index, which is simply the decile of socio-economic advantage from the lowest to the highest ten percent. The fact that the relative frequencies all approximate to the value of ten gives an indication of the representativeness of the HILDA sample. The final column gives the distribution of scores over the five panel years for the BMI categories. Table 3 complements this column by giving the transition probabilities between categories between the first and last years of the sample.

Reading down the columns in Table 3 gives the BMI category in 2006, and reading across the rows gives the category in 2010. The elements on the principal diagonal give the probability of remaining in the same category: these are relatively large, indicative of stability over time. The off-diagonal elements give the probability of transition between categories. The probability of moving from *normal* to *overweight* is 0.152 and the probability of moving from *obese* to *normal* is 0.016. These are unconditional transitional probabilities. The next section examines the conditional probabilities of being overweight or obese, identified by the random effects generalized ordered probit model.

## 4. Results

The random effects specification was applied to Equation (6), and this model was estimated without restriction. Recalling Section 2, a series of sequential Wald tests were applied to this unrestricted model, where each test is on the basis of the parallel lines restriction to variables. The variable with the highest probability value was then restricted and the model re-estimated with the restriction, with the subsequent imposition of the parallel lines restriction onto the remaining variables. This process of test and then restriction proceeded until only those variables with a probability score of less than 0.05 in the Wald test remained. See Table A1 of the Appendix A for the probability scores of the sequential Wald tests for all variables. Six variables were identified where the parallel lines restriction applied. That is, the estimated coefficients for these variables were deemed to be the same for both equations in the model. Table 4 gives a Wald test on jointly restricting these variables to have the same coefficient values over the two equations and clearly indicates that these restrictions cannot be dismissed. 

The results for the generalized ordered probit of Equation (6) from Section 2, but estimated with the parallel lines assumption of Table 4, are given in Table 5. The results for the generalized ordered probit without parallel lines restrictions are reported without comment in Table A2 of the Appendix A. The first two columns of Table 5 give the coefficients and standard errors for eq1, with the category *normal* in the variable *ordobese*, against the two categories *overweight* and *obese*. The following two columns of coefficients and standard errors are for eq2, with the categories *normal* and *overweight* against the category *obese*. The underlined coefficients are for those variables where the parallel lines restriction is not relaxed according to the test outcomes of Table 4. It should be noted that the coefficients for three of these variables, *hhtype*, *alcohol*, and *meals*, test as not significantly different from zero in both eq1 and eq2. Further, the estimates of the unrestricted generalized ordered probit, as shown in Table A2 of the Appendix A, show that the remaining three variables with parallel line restrictions, *area*, *agree*, and *emote*, have estimated coefficients which are all significant at the 1% level in both equations and have similar magnitudes in both equations for the unrestricted estimates. 

Before moving on to a detailed description of the estimated coefficients and their implications for the BMI categories, it would be useful to deal with two statistics reported in the header and footer of Table 5. The Wald test at the header of the table is the usual model test with the slope parameters jointly restricted to zero. Note here that there are 30 slope parameters, as the model is estimated conditional on six parameters being common to both eq1 and eq2. In the footer of the table, *ρ* is the ratio $\rho =\sigma_{\alpha }^{2}/(\sigma_{\alpha }^{2}+\sigma_{\varepsilon }^{2})$ where $\sigma_{\alpha }^{2}$ and $\sigma_{\varepsilon }^{2}$ are the variance of the unobserved individual effect and the idiosyncratic error, respectively. The statistic *ρ* is restricted to the unit interval and it gives the proportion of the total variance given by the unobserved individual effect. It is the correlation of *overweight* and *obese* over time for individuals and is indicative of the level of persistence in the *overweight*/*obese* category against *normal* weight and the *obese* category against the *normal*/*overweight* category for individuals [48,49]. Here the score, at 0.852, is close to 1.0 and is indicative of high persistence for individuals over time. 

Ten variables are significant at the 1% level in both equations. Of these, three are restricted to having fixed coefficients for both equations, *area*, *agree*, and *emote*. Remoteness area, *area*, is ordered categorically, and the ordering is over increasing remoteness. Thus probability in both equations is increasing with remoteness. Recall that the personality scores are coded from 1 to 7, with low scores reflecting negative aspects of the trait and high scores reflecting positive aspects of the trait. Increasing emotional stability, *emote*, is associated with lower probability, but the reverse is true for agreeableness, *agree*, with increasing agreeableness indicating a higher probability.

The seven variables for which the parallel lines restriction does not apply and which are significant in both equations are *age*, *age2*, *educ*, *advantage*, *marstatus*, *consc*, and *opene*. The outcomes for *age* and *age2* indicate that probability is increasing, but this is non-linear in both equations. The education variable, *educ*, is an ordinal scale in qualification achieved, ranked from highest to lowest. The positive sign is expected and probability is decreasing with educational achievement [39,40]. The ordinal scale for *advantage*, the SEIFA deciles of relative socio-economic advantage, is increasing and the negative sign is expected. Being in a married or de facto relationship, *marstatus*, shifts the probability up in both equations. A rationale for this may be in terms of marriage markets; once married, competition for a partner ceases, and individuals become less concerned with appearance. That is, marriage causes overweightness. However, this also opens up the possibility of marriage selection and that lean people are more likely to be selected in marriage [50], although Lin et al. found some evidence to suggest that when single females are faced with adverse marriage market conditions, low male to female ratios, then females have less incentive to remain fit and healthy [51]. The discussion of the two personality traits, *consc* and *opene*, will be deferred to a joint examination of the implications of the results for all five psychological variables.

Two variables are significant in eq2 but not in eq1. There is a significant downward shift in the probability of being *obese* relative to falling into the *normal/overweight* category that is associated with being employed, *empstatus*. The categorical variable *losat* is positively ordered in life satisfaction; that is, higher scores indicate increased life satisfaction. The outcome of a negative relationship with probability in eq2 is expected. Three variables are significant in eq1, but not in eq2; *gender*, *lndinc/p*, and *extrv*. All three coefficients are positive, so that probability increases in eq1 but not in eq2. The results indicate that being male increases the likelihood of being overweight. Further, the likelihood of being overweight increases with household disposable income per person, *lndinc/p*.

All of the FFM personality traits are significant with the exception of extraversion in eq2. Three have negative coefficients; *consc*, *emote*, and *opene*. That is, the probability of being overweight/obese is decreasing with increasing conscientiousness, emotional stability, and openness to ideas. Given the nature of these traits, the negative sign would be expected. Similar findings on the association between BMI and body weight and conscientiousness were revealed by Kim (2016). That study used participants from the National Longitudinal Study of Adolescent to Adult (Add Health) and concluded that a one standard deviation increase in conscientiousness was associated with a decrease in BMI by 0.89 and a 12% reduction in the probability of being obese [21]. There is nothing in the nature of the remaining traits, agreeableness and extraversion, which would indicate any conditioning of probability. However, the results here replicate the results for the community in Sullivan et al. [20]. They found that being obese was positively associated with novelty seeking and reduced reward dependence, which is parallel with the positive sign for extraversion here. Further, the negative signs for conscientiousness and emotional stability are replicated in Sullivan et al., with lower persistence and self-directedness being associated with obesity [20]. 

This study had a number of limitations. It relied on, and analysis was based on, HILDA panel data without any augmentation to link to other datasets for one or more periods, such as health campaigns at national or state level. In addition, within the HILDA dataset, households may have been comprised by one or many more than one person, but no attempt was made in the analysis to group respondents by household. A third limitation was the exclusion of “underweight” due to the small proportion of respondents in this category, and the grouping of persons with BMI > 30 into the *obese* category with no further attempt to include another category of *morbidly obese*.

## 5. Conclusions

A generalized ordered probit model was estimated to identify those factors that influence being overweight or obese relative to normal weight. The parallel lines assumption of the standard ordered probit model could not be dismissed for six of the 18 conditioning variables, but where two of these did not have coefficients, it significantly different from zero. This suggests that the generalized order probit, with partial restriction, is the appropriate specification. The probability of being overweight/obese relative to normal weight and the probability of being obese relative to normal/overweight was found to be conditioned by 13 and 12 of the 18 candidate variables, respectively. The personality traits of the FFM were included in the set of covariates to control for a predisposition to being obese. These personality traits were found to be significant in conditioning probability and replicated outcomes of another study where different traits, but ones that correlated to the FFM were used. This finding may have important implications. If personality traits are indicative of a predisposition to being obese, then any policy mechanism designed to combat obesity must take this into account [27]. Policies which concentrate on lifestyle choice and economic and social factors may be inefficient if the relationship between obesity and personality is ignored. 

## Figures and Tables

**Table 1:** Description and descriptive statistics for the covariates used in the model.

Covariates Name	Description	Mean (Std Dev.)
*ordobese*	Body mass index group (BMI); Normal = 1, Overweight = 2, Obese = 3	2.810 (0.780)
*gender*	Male = 1, Female = 0	0.476 (0.500)
*age*	Age in years	45.866 (17.848)
*age*2	Square of age	2422.212 (1734.891)
*empstatus*	Labour force status; employed = 1, unemployed and not in the labour force = 0	0.658 (0.474)
*area*	Remoteness area; major city = 0, inner regional Australia = 1, outer regional Australia = 2, remote Australia = 3, very remote Australia = 4	0.535 (0.777)
*hhtype*	Household type; lone parent with children < 15 = 1, others = 0	0.304 (0.181)
*losat*	Satisfaction with life; dissatisfied = 1, neutral = 2, satisfied = 3	2.877 (0.361)
*lndinc/p*	Logarithm of the ratio of household disposable income to the number of persons in household.	10.108 (0.640)
*alcohol*	SCQ—Household annual expenditure on alcohol (A$)	1450.713 (2355.439)
*meals*	SCQ—Household annual expenditure on meals eaten out (A$)	2443.758 (2809.403)
*educ*	Highest education level achieved; Postgrad-Bachelor = 1, Adv. Diploma-Certificate = 2, Year 12 = 3, Year 11 and below = 4	2.528 (1.152)
*advantage*	SEIFA 2001 index by decile of relative socio-economic advantage/disadvantage; lowest = 1, highest = 10	5.623 (2.869)
*marstatus*	Marital status; married or in a de facto relationship = 1, single = 0	0.658 (0.474)
*agree*	Personality scale—Agreeableness (1 to 7)	5.369 (0.912)
*consc*	Personality scale—Conscientiousness (1 to 7)	5.105 (1.020)
*emote*	Personality scale—Emotional stability (1 to 7)	5.234 (1.068)
*extrv*	Personality scale—Extroversion (1 to 7)	4.429 (1.068)
*opene*	Personality scale—Openness to experience (1 to 7)	4.188 (1.057)

**Table 2:** The frequency distribution of categorical variables (percent).

Categories/Variables	*Advantage*	*Educ*	*Area*	*Losat*	*Ordobese*
0			61.69		
1	8.51	23.31	25.28	1.11	41.68
2	10.24	31.16	11.19	10.12	35.59
3	10.46	14.96	1.5	88.78	22.72
4	9.44	30.58	0.34		
5	9.99				
6	9.35				
7	9.96				
8	10.58				
9	11.00				
10	10.47				

**Table 3:** Transition probabilities of BMI groups.

Year	BMI Category	2010		
		Normal	Overweight	Obese
**2006**	**Normal**	0.838	0.152	0.009
	**Overweight**	0.138	0.748	0.114
	**Obese**	0.016	0.148	0.836

**Table 4:** Wald test of parallel lines assumption.

H_0_:
eq1 *meals* − eq2 *meals* = 0;
eq1 *area* − eq2 *area* = 0;
eq1 *alcohol* − eq2 *alcohol* = 0;
eq1 *agree* − eq2 *agree* = 0;
eq1 *emote* − eq2 *emote* = 0; and
eq1 *hhtype* − eq2 *hhtype* = 0
H_1_:
Not H_0_
Wald *χ*^2^(6) = 3.81
Pr > *χ*^2^ = 0.702

**Table 5:** Estimates for the restricted generalized order probit.

Number of Observations = 46,909				
Log Likelihood = −31,495.55				
Wald *χ*^2^(30) = 2415.36; Pr > *χ*^2^ = 0.0000				
Covariates	eq1		eq2	
	Coef.	Std Err.	Coef.	Std Err.
*gender*	0.8837	0.0512 ***	0.0620	0.0529
*age*	0.1905	0.0071 ***	0.1432	0.0080 ***
*age*2	−0.0016	0.0001 ***	−0.0013	0.0001 ***
*empstatus*	−0.0203	0.0379	−0.1500	0.0423 ***
*area*	0.0790	0.0251 ***	0.0790	0.0251 ***
*hhtype*	0.0447	0.0732	0.0447	0.0732
*losat*	0.0537	0.0358	−0.1123	0.0376 ***
*lndinc/p*	0.1132	0.0250 ***	0.0173	0.0265
*alcohol*	−5.34E-06	5.18E-06	−5.34E-06	5.18E-06
*meals*	1.32E-06	4.28E-06	1.32E-06	4.28E-06
*educ*	0.1160	0.0226 ***	0.1682	0.0247 ***
*advantage*	−0.0850	0.0075 ***	−0.1096	0.0078 ***
*marstatus*	0.3846	0.0419 ***	0.2161	0.0454 ***
*agree*	0.0920	0.0179 ***	0.0920	0.0179 ***
*consc*	−0.1555	0.0192 ***	−0.1956	0.0205 ***
*emote*	−0.0572	0.0158 ***	−0.0572	0.0158 ***
*extrv*	0.0623	0.0176 ***	0.0100	0.0187
*opene*	−0.0939	0.0190 ***	−0.0572	0.0203 ***
*constant*	−5.2483	0.3632 ***	−4.4469	0.3789 ***
*ρ*	0.852			

*** 1% significance.

## Appendix A

Table A1 gives the results of the sequential parameter restriction tests to identify those variables to which the parallel lines restriction may apply. Note that the chosen probability is 0.05. It should also be noted that had the 0.1 level been used, the number of restrictions would have been the same.

**Table A1:** Sequential parameter restriction test.

Variable	Pr
*meals*	0.9935 *****
*area*	0.7686 *****
*alcohol*	0.5540 *****
*agree*	0.4177 *****
*emote*	0.2876 *****
*hhtype*	0.2084 *****
*gender*	0.00000
*age*	0.00000
*age*2	0.00000
*empstatus*	0.00430
*losat*	0.00015
*lndinc/p*	0.00125
*educ*	0.00591
*advantage*	0.00060
*marstatus*	0.00007
*consc*	0.03451
*extrv*	0.00278
*opene*	0.04357

***** Apply parallel lines restriction.

The results for the unrestricted model are given in Table A2. The structure of this table is identical to that of Table 5 in the text. The estimates of Table A2 are very similar to the restricted model and this could be taken as indicative of a relatively robust estimate.

**Table A2:** Estimates for the unrestricted generalized order probit.

Number of Observations = 46,909				
Log Likelihood = −31,493.659				
LR *χ*^2^(36) = 32,174.10; Pr > *χ*^2^ = 0.0000				
Covariates	eq1		eq2	
	Coef.	Std Err.	Coef.	Std Err.
*gender*	0.8844	0.0513 *******	0.0596	0.0535
*age*	0.1904	0.0071 *******	0.1422	0.0081 *******
*age*2	−0.0016	0.0001 *******	−0.0013	0.0001 *******
*empstatus*	−0.0200	0.0379	−0.1464	0.0423 *******
*area*	0.0821	0.0275 *******	0.0745	0.0297 *******
*hhtype*	−0.0085	0.0843	0.1075	0.0871
*losat*	0.0520	0.0358	−0.1076	0.0377 *******
*lndinc/p*	0.1105	0.0252 *******	0.0221	0.0269
*alcohol*	−3.25E-06	6.40E-06	−8.30E-06	7.54E-06
*meals*	1.22E-06	5.14E-06	1.28E-06	5.96E-06
*educ*	0.1145	0.0227 *******	0.1658	0.0248 *******
*advantage*	−0.0848	0.0077 *******	−0.1094	0.0078 *******
*marstatus*	0.3768	0.0423 *******	0.2222	0.0463 *******
*agree*	0.0996	0.0204 *******	0.0808	0.0219 *******
*consc*	−0.1577	0.0194 *******	−0.1890	0.0209 *******
*emote*	−0.0497	0.0179 *******	−0.0688	0.0193 *******
*extrv*	0.0604	0.0178 *******	0.0127	0.0189
*opene*	−0.0937	0.0193 *******	−0.0565	0.0209 *******
*constant*	−5.2703	0.3657 *******	−4.4215	0.3834 *******

******* 1% significance.
